# Natural course of visual snow syndrome: a long-term follow-up study

**DOI:** 10.1093/braincomms/fcac230

**Published:** 2022-09-09

**Authors:** Michael Graber, Adrian Scutelnic, Antonia Klein, Francesca Puledda, Peter J Goadsby, Christoph J Schankin

**Affiliations:** Department of Neurology, Inselspital, Bern University Hospital, University of Bern, Bern 3010, Switzerland; Department of Neurology, Inselspital, Bern University Hospital, University of Bern, Bern 3010, Switzerland; Department of Neurology, Inselspital, Bern University Hospital, University of Bern, Bern 3010, Switzerland; Headache Group, Wolfson CARD, Institute of Psychiatry, Psychology and Neuroscience, King’s College London SE1 1YR, London, UK; NIHR-Wellcome Trust King’s Clinical Research Facility, SLaM Biomedical Research Center, King’s College London SE5 9PJ, London, UK; Headache Group, Wolfson CARD, Institute of Psychiatry, Psychology and Neuroscience, King’s College London SE1 1YR, London, UK; NIHR-Wellcome Trust King’s Clinical Research Facility, SLaM Biomedical Research Center, King’s College London SE5 9PJ, London, UK; Department of Neurology, Inselspital, Bern University Hospital, University of Bern, Bern 3010, Switzerland

**Keywords:** visual snow, migraine, aura, natural course, follow-up

## Abstract

Visual snow syndrome is characterized by a continuous visual disturbance resembling a badly tuned analogue television and additional visual and non-visual symptoms causing significant disability. The natural course of visual snow syndrome has not hitherto been studied. In this prospective longitudinal study, 78 patients with the diagnosis of visual snow syndrome made in 2011 were re-contacted in 2019 to assess symptom evolution using a semi-structured questionnaire. Forty patients (51% of 78) were interviewed after 84 ± 5 months (mean ± SD). In all patients, symptoms had persisted. Visual snow itself was less frequently rated as the most disturbing symptom (72 versus 42%, P = 0.007), whereas a higher proportion of patients suffered primarily from entopic phenomena (2 versus 17%, P = 0.024). New treatment was commenced in 14 (35%) patients, of whom in seven, visual snow syndrome was ameliorated somewhat. Three (7%) experienced new visual migraine aura without headache, and one (2%) had new migraine headache. There were no differences in the levels of anxiety and depression measured by the Patient Health Questionnaire 8 and the Generalized Anxiety Disorder Scale 7. Thirty-eight patients (49%) were lost to follow-up. In visual snow syndrome, symptoms can persist over 8 years without spontaneous resolution, although visual snow itself might become less bothersome.

## Teaser

Graber *et al*. report first longitudinal data on visual snow syndrome. Symptoms persisted throughout the duration of the study (mean 7 years). In several patients, enhanced entoptic phenomena replaced visual snow itself as the most bothersome symptom. Most patients with isolated continuous visual snow developed additional symptoms over time.

## Graphical Abstract

**Graphical abstract:**
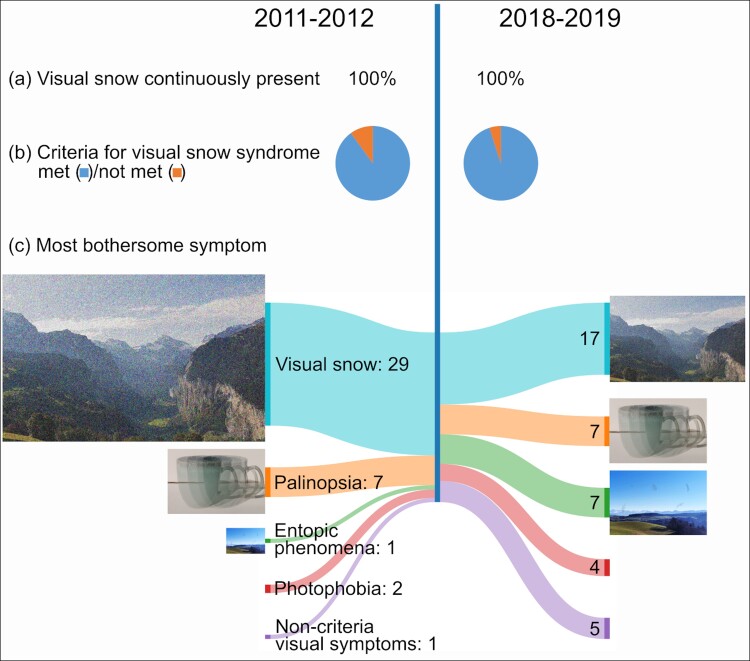

## Introduction

Patients with ‘visual snow syndrome’ (VSS) suffer from a visual disturbance resembling the static of a badly tuned analogue television (visual snow) and additional visual symptoms, such as palinopsia, photophobia, nyctalopia, and enhanced entopic phenomena.^1^ Approximately 40% of patients report experiencing visual snow since early childhood.^2^

Little is known about the natural course of continuous visual snow or of the syndrome VSS. Retrospective data based on interview^1^ and online surveys^2^ suggest that symptoms can be present over several years, but longitudinal data is lacking. The aim of our study was to assess prospectively the evolution of the visual symptoms of VSS, some comorbidities, and treatment response in the same patient population over time.

## Materials and methods

This longitudinal prospective study has been approved by the local ethics committee (KEK 2018-01376). Preliminary data have been presented at the 6th Congress of the European Academy of Neurology (Virtual, 23–26 May 2020).^3^

### Patients

We attempted to re-contact the 78 patients with continuous visual snow and normal ophthalmological exams from the first systematic study that took place between November 2011 and March 2012^1^ and eventually resulted in the definition of criteria for VSS in the appendix of the International Classification of Headache Disorders, 3rd edition (ICHD-3).^4^ All patients had agreed to be contacted for potential follow-up studies.

### Telephone interview

Patients were contacted between September 2018 and November 2019 using the last available contact information. At least three attempts were made using email, or telephone, or both. When patients could be reached and agreed to participate in the study, telephone interviews were performed by experts in neurology and headache (M.G. and A.S.). Using the semi-structured questionnaire from the first study^1^ and encouraging the patients to use their own descriptive wording, the following parameters were assessed:

- current visual symptoms with a focus on the most disturbing symptom,
- aggravating and relieving factors,
- new visual symptoms since the last interview,
- interrogation of the individual criteria for VSS,^1^
- intake of illicit drugs prior to any worsening/onset/recurring of visual snow,
- investigations performed to explain visual snow,
- new onset of headache and, if applicable, classification of new headache according to the ICHD-3,^4^
- new onset of migraine aura,^4^
- new onset of other medical problems,
- medical treatments and its effects on visual snow since the first interview; it was specified whether the treatments were commenced specifically for visual snow or for other conditions, and
- Patient Health Questionnaire (PHQ)-8^5^ for depression and Generalized Anxiety Disorder (GAD-7)^6^ for anxiety. The threshold for moderate or severe depression was a PHQ-8 score of ≥10 points. Moderate to severe anxiety was defined as a GAD-7 score of ≥10 points.

As we know now, VSS is a complex disorder with additional non-visual and non-perceptual symptoms.^7^ For a direct comparability with the initial study,^1^ however, the other symptoms such as vertigo, depersonalization, disrupted sleep, and fatigue^7^ were not specifically addressed here.

### Statistical analysis

SPSS (v25, IBM Corp.) was used for data analysis. Chi-square or Fisher’s exact test was used for categorical variables. Standard descriptive statistics were applied with counts, frequencies and means with standard deviations. Significance was assumed at a *P*-value of <0.05.

## Results

Forty patients (51% of 78) could be reached and agreed to participate in the study. Mean age was 38 (range 18–63), and 19 (47% of 40) were female. Despite several attempts, 38 (49% of 78) patients were lost to follow-up. Mean time ± SD passed between initial interview and follow-up was 83 ± 5 months. Twelve (30% of 40) patients had visual snow for as long as they could remember. In the remaining 28 (70% of 40), mean age of onset ± SD was 22 ± 11 years. At the time of follow-up, patients had been suffering from visual snow for 23 ± 15 years. Four (10%) had first-grade relatives suffering from visual snow.

### Clinical change over time

One patient (2%) reported isolated visual snow, the remainder 39 (98%) had additional visual symptoms (Table 1). Compared with the initial interview, two additional patients fulfilled criteria for VSS at follow-up [38 (94%) versus 36 (90%)]. One patient stated loss of peripheral vision on both eyes as well as new onset of after-images, and the other experienced spontaneous photopsia in the peripheral vision. Three patients (7%) experienced new entopic phenomena during follow-up compared with baseline [36 (90%) versus 33 (83%)]. One (2%) patient had new photophobia [25 (62%) versus 24 (60%)]. Three patients (7%) reported improvement of palinopsia and trailing [30 (75%) versus 33 (82%)] and two (5%) cessation of nyctalopia [22 (55%) versus 24 (60%)].

**Table 1:** Visual symptoms at baseline and during follow-up

VS criteria	Baseline, *n*/*N* (%)	Follow-up, *n*/*N* (%)	*P*-value, (*χ*^2^)
Visual snow	40/40 (100)	40/40 (100)	1
Entoptic phenomena	33/40 (82)	36/40 (90)	0.33
Palinopsia or trailing	33/40 (82)	30/40 (75)	0.412
Photophobia	24/40 (60)	25/40 (62)	0.818
Nyctalopia	24/40 (60)	22/40 (55)	0.651
Visual snow syndrome criteria fulfilled	36/40 (90)	38/40 (94)	0.396

At follow-up, visual snow did not remain the most disturbing symptom in 12 (30%) patients corresponding to a reduction from 29 (72%) to 17 (42% of 40, *P* = 0.007, Pearson *χ*^2^ = 7.36, Table 2). In contrast, 6 (15%) patients reported entopic phenomena to be most disturbing corresponding to an increase from 1 (2%) to 7 of 40 (17%, *P* = 0.025, Pearson *χ*^2^ = 5.0), of whom 4 reported floaters, 1 photopsia, and 1 self-light of the eye. At follow-up, four (10% of 40) patients reported most disturbance from visual symptoms not part of the VSS criteria [increase from one (2%) to five (12%)]. Symptoms were as follows: monocular double vision, eye strain with accommodation issues, tunnel vision, and difficulties looking at bright lights.

**Table 2:** Most disturbing visual symptom at follow-up

Most disturbing symptom	1st interview (2011–12), *n*/*N* (%)	2nd interview (2018–19), *n*/*N* (%)	*P*-value (*χ*^2^ or Fisher’s exact test)
Visual snow	29/40 (72)	17/40 (42)	0.007
Palinopsia or trailing	7/40 (17)	7/40 (17)	1
Entoptic phenomena	1/40 (2)	7/40 (17)	0.025
Floaters	1/40 (2)	5/40 (12)	0.090
Photopsia	0/40 (0)	1/40 (2)	0.310
Self light of the eye	0/40 (0)	1/40 (2)	0.310
Photophobia	2/40 (5)	4/40 (10)	0.396
Nyctalopia	0/40 (0)	0/40 (0)	–
Non-criteria visual symptoms	1/40 (2)	5/40 (12)	0.090

### Changes associated with migraine

Nineteen (47%) patients met criteria for migraine, of whom one reported new onset since the last interview. Fourteen (35%) reported typical migraine visual aura, of whom three (21%) described new occurrence of migraine aura at follow-up.

### Investigations over time for VSS

Investigations performed specifically for VSS were done in 11 (27%) patients: 7 had magnetic resonance imaging (MRI), 1 spinal MRI, 4 electroencephalography (EEG), 1 visual evoked potentials, 1 computed tomography, and 5 ophthalmologic examination, all of which were unremarkable or non-contributory. In a patient with confirmed mitochondrial encephalopathy with carnitine deficiency, an EEG showed intermittent slowing and the head MRI signs of unspecific leukoencephalopathy. The same patient underwent neuropsychological evaluation, which revealed abnormalities in short term memory, as well as muscle biopsy and needle electroneuromyography, both with unremarkable results.

### Depression and anxiety over time

At baseline, GAD-7 and PHQ-8 questionnaires have been conducted in 33 patients. One additional patient fulfilled criteria for moderate or severe depression at follow-up compared with baseline [5/33 (15%) versus 4/33 (12%)]. Five patients (15%) had moderate to severe anxiety at follow-up, unchanged from baseline.

### Treatments

Fourteen patients (35% of 40) tried treatment and/or lifestyle modifications for visual snow. Seven (50% of 14) reported some amelioration, but no cessation of the visual symptoms (Table 3). Among pharmacological treatments, magnesium, cinnarizine, and flunarizine had been beneficial. Two patients reported improvement of VSS after lamotrigine, verapamil, lorazepam and nortriptyline intake, prescribed for prevention of migraine, whereas metronidazole, botulinum toxin Type A, statins, and topiramate lead to worsening of visual symptoms.

**Table 3:** Treatments with influence on visual symptoms

Age of onset of visual snow	Treatment tried	Influence on visual symptoms (yes/no)	Co-existing migraine
52	Topiramat, gluten-free diet, fish oil, supplemental vitamin D and co-enzyme Q intake	Yes, topiramat worsened the symptoms; fish oil, supplemental vitamin D and co-enzyme Q intake alleviated	No
10	Verapamil and nortriptyline	Yes, alleviation	Yes
35	Valproate	Yes, alleviation	Yes
13	Chiropractic treatment	Yes, temporary alleviation for two weeks	No
20	Botulinum toxin Type A injections for migraine prophylaxis	Yes, worsening	Yes
40	Polarized glasses	Yes, alleviation of light sensitivity	Yes
21	Gabapentine, lamotrigine, baclofen, Acetazolamid, lorazepam	Yes, lorazepam alleviated the symptoms; all other treatment worsened them	No
22	Magnesium	Yes, alleviation	Yes
33	Flunarizin, cinnarizine	Yes, alleviation after both medications	Yes
22	Metronidazole	Yes, worsening	No

Chiropractic cervical adjustment leads to improvement in one patient. Other successful treatments were gluten-free diet, fish oil, supplemental vitamin D and co-enzyme Q intake, and wearing polarized glasses.

Seven patients reported new diagnoses at follow-up (Table 4).

**Table 4:** New comorbidities identified at follow-up

Age of onset of visual snow	Age at diagnosis (years)	New comorbidity
10	38	Intestinal polyps
14	35	Lyme disease, chronic active Epstein-Barr virus infection, small fibre neuropathy, chronic fatigue syndrome
Since birth	57	Rosacea, syndrome of intestinal bacterial overgrowth
24	32	Depression, anxiety
Since birth	31	Attention deficit and hyperactivity disorder, endometriosis, polycystic ovary syndrome, Hashimoto thyroiditis
30	39	Syndrome of intestinal bacterial overgrowth, chronic pancreatitis, mitochondrial encephalopathy
52	63	Metabolic syndrome

## Discussion

The main findings of our study are as follows: (i) over a mean of 7 years no participant with visual snow went into spontaneous complete remission; (ii) over time, entopic phenomena might replace visual snow as their most bothersome symptom; (iii) patients with visual snow as their sole symptom might worsen over time finally meeting criteria for VSS; and (iv) there are currently no reported lifestyle or therapeutic measures leading to complete cessation of VSS, although modest improvement is possible.

Our data confirms prospectively that having visual snow in the non-episodic form^8^ means that symptoms will persist over several more years as suggested so far only from previous cross-sectional studies.^1,2^ Furthermore, patients continued to fulfil criteria for VSS at follow-up. Of four patients who did not fulfil the criteria at baseline, three developed new visual symptoms, which resulted in the diagnosis of VSS in two patients. This supports that the underlying process can be progressive. The continuing lack of pathologic findings in the ancillary examinations suggest that VSS might be a primary brain disorder, analogous to the primary headache disorders of the ICHD-3.^9,10^ Indeed, several patients who did not fulfil criteria for migraine with or without aura in the initial study developed migraine over the course of the following years. This emphasizes the close interconnection between migraine and VSS.^7^ Almost half (47%) of patients included in this study suffer from migraine headache, and a third (35%) from migraine with aura, in contrast to the much lower prevalence in the general population (20 and 6%, respectively^11,12^). There also appears to be an interconnection between severity of VSS and comorbid migraine.^13^

The reason for entoptic phenomena replacing visual snow itself as the most disturbing symptom remains unclear. It is possible that the continuing perception of a noise-like visual disturbance can be ignored more easily than enhanced entopic phenomena, which occur only under specific circumstances, such as blue field entoptic phenomena, or with changing shape or drifting location, such as floaters. Remarkably, the other visual symptoms remained constant regarding severity, and only few patients reported occurrence or cessation of isolated symptoms over time. This supports the validity of the initial proposed diagnostic criteria for VSS^1^ and the stability of the mechanism causing the entire syndrome.

The improvement without cessation of VSS with certain changes of lifestyle and medication is in line with previous retrospective reports.^14^ The effect of lamotrigine and verapamil in only few patients has been reported.^14^ However, efficacy of cinnarizine, flunarizine, and nortriptyline in VSS has not been reported yet. The potential benefit from benzodiazepines reported previously from an online survey is supported by our patient who improved from lorazepam.^15^ In contrast to previous reports, one patient described worsening of symptoms after intake of topiramate.^12,16^ It is worth mentioning that some patients experienced worsening of symptoms after treatment with statins and botulinum toxin Type A. Most patients who experienced alleviation of visual symptoms had comorbid migraine (Table 3). It is thus possible that alterations were mediated by migraine modulation since most medications might have some effect on migraine, at least in part. General conclusions from this data on treatment cannot be drawn, since these findings are anecdotal and the level of evidence is low.

Our study has limitations. Firstly, a high proportion of patients were lost to follow-up, making selection bias possible. Secondly, the overall number of patients included in this study is low limiting generalizability. Thirdly, we cannot exclude recall bias, given the large time-span between the baseline and follow-up interview. Such bias regarding the course of the symptoms is unlikely, given the persistent nature of the symptoms. However, details regarding the dosage and duration of intake of medication, performed investigations, and new diagnoses might have been more influenced by a recall bias. Fourthly, we cannot exclude suggestibility of answers during the interview. However, patients were given the possibility to provide an open answer after every question. Finally, it has to be emphasized that VSS is a complex disorder extending beyond the visual system.^7^ For direct comparability with the initial paper,^1^ the design of our study focused on the visual symptoms. As we did not prospectively evaluate the non-visual symptoms, such as vertigo, depersonalization, disrupted sleep, or fatigue, future prospective studies are necessary to better understand the clinical course of the entire disorder.

Our study has also strengths. It is the largest cohort of VSS patients from whom follow-up data are available. The interviews were performed by trained neurologists, minimizing the possibility of misinterpreting the questions.

## Conclusion

Although the symptoms persist over years, VSS patients experience a change in the relative burden of individual symptoms. Knowing the natural history will be essential for daily communication with our patients. Telling them that ‘waiting for the symptoms to disappear’ will not be an option. Currently, no treatment or lifestyle measures lead to cessation of VSS underscoring the necessity to better understand the mechanism of the condition. Most patients are forced to wait until a successful treatment is found.

## Data Availability

Anonymized data will be shared upon reasonable request.

## Abbreviations

GAD =: Generalized Anxiety Disorder Scale
ICHD-3 =: International Classification of Headache Disorders 3rd edition
PHQ =: Patient Health Questionnaire
VSS =: Visual snow syndrome
